# DNA methylation and cardiovascular disease in humans: a systematic review and database of known CpG methylation sites

**DOI:** 10.1186/s13148-023-01468-y

**Published:** 2023-03-30

**Authors:** Mykhailo Krolevets, Vincent ten Cate, Jürgen H. Prochaska, Andreas Schulz, Steffen Rapp, Stefan Tenzer, Miguel A. Andrade-Navarro, Steve Horvath, Christof Niehrs, Philipp S. Wild

**Affiliations:** 1 Preventive Cardiology and Preventive Medicine, Department of Cardiology, University Medical Center of the Johannes Gutenberg University Mainz, Langenbeckstr. 1, 55131 Mainz, Germany; 2 Institute of Molecular Biology (IMB), 55128 Mainz, Germany; 3 Division of Molecular Embryology, DKFZ-ZMBH Alliance, 69120 Heidelberg, Germany; 4 Systems Medicine, Institute of Molecular Biology (IMB), Ackermannweg 4, 55128 Mainz, Germany; 5 Clinical Epidemiology and Systems Medicine, Center for Thrombosis and Hemostasis (CTH), Mainz, Germany; 6 German Center for Cardiovascular Research (DZHK), Partner Site Rhine Main, University Medical Center of the Johannes Gutenberg University Mainz, Mainz, Germany; 7 Altos Labs, San Diego, USA; 8 Institute for Immunology, University Medical Center of the Johannes Gutenberg-University Mainz, Mainz, Germany; 9 Institute of Organismic and Molecular Evolution, Johannes Gutenberg-University Mainz, Mainz, Germany

**Keywords:** Epigenetics, DNA methylation, Cardiovascular, CpG, Systematic review, CVD, Heart, Vascular

## Abstract

**Background:**

Cardiovascular disease (CVD) is the leading cause of death worldwide and considered one of the most environmentally driven diseases. The role of DNA methylation in response to the individual exposure for the development and progression of CVD is still poorly understood and a synthesis of the evidence is lacking.

**Results:**

A systematic review of articles examining measurements of DNA cytosine methylation in CVD was conducted in accordance with PRISMA (preferred reporting items for systematic reviews and meta-analyses) guidelines. The search yielded 5,563 articles from PubMed and CENTRAL databases. From 99 studies with a total of 87,827 individuals eligible for analysis, a database was created combining all CpG-, gene- and study-related information. It contains 74,580 unique CpG sites, of which 1452 CpG sites were mentioned in ≥ 2, and 441 CpG sites in ≥ 3 publications. Two sites were referenced in ≥ 6 publications: cg01656216 (near *ZNF438*) related to vascular disease and epigenetic age, and cg03636183 (near *F2RL3*) related to coronary heart disease, myocardial infarction, smoking and air pollution. Of 19,127 mapped genes, 5,807 were reported in ≥ 2 studies. Most frequently reported were *TEAD1* (TEA Domain Transcription Factor 1) and *PTPRN2* (Protein Tyrosine Phosphatase Receptor Type N2) in association with outcomes ranging from vascular to cardiac disease. Gene set enrichment analysis of 4,532 overlapping genes revealed enrichment for Gene Ontology molecular function “DNA-binding transcription activator activity” (*q* = 1.65 × 10^–11^) and biological processes “skeletal system development” (*q* = 1.89 × 10^–23^). Gene enrichment demonstrated that general CVD-related terms are shared, while “heart” and “vasculature” specific genes have more disease-specific terms as PR interval for “heart” or platelet distribution width for “vasculature.” STRING analysis revealed significant protein–protein interactions between the products of the differentially methylated genes (*p* = 0.003) suggesting that dysregulation of the protein interaction network could contribute to CVD. Overlaps with curated gene sets from the Molecular Signatures Database showed enrichment of genes in hemostasis (*p* = 2.9 × 10^–6^) and atherosclerosis (*p* = 4.9 × 10^–4^).

**Conclusion:**

This review highlights the current state of knowledge on significant relationship between DNA methylation and CVD in humans. An open-access database has been compiled of reported CpG methylation sites, genes and pathways that may play an important role in this relationship.

**Supplementary Information:**

The online version contains supplementary material available at 10.1186/s13148-023-01468-y.

## Introduction

Cardiovascular disease (CVD) is the leading cause of death globally, accounting for approximately 30% of all deaths worldwide [1]. This puts an enormous burden on healthcare systems across the globe and has prompted clinicians and researchers to explore potential causes of this widespread disease. The World Health Organization recognizes CVD as one of the most environmentally driven diseases with a comparatively small genetic component [2]. It is likely that epigenetic changes mediate, at least in part, the environmental risk for developing or progressing CVD. One prominent factor that is thought to play a role is DNA methylation, an epigenetic mark that can modify gene expression. DNA methylation usually refers to the methylation of the fifth carbon of cytosine residues found throughout the genome. Methylation is most commonly observed at so-called CpG islands [3], which are long repeats of cytosine-guanine nucleotides. As technologies to study genome-wide DNA methylation continue to develop, there is growing evidence that there is a strong link between DNA methylation and CVD [4–6]. However, there is still little to no evidence of the directionality of this relationship, and clear findings on relevant CpG sites or genes have not yet emerged from the extensive research in this area.

The main aim of this systematic review was to synthesize results of studies that have investigated the relationship between DNA methylation and CVD and to create an easily accessible and searchable database from the results of these studies. Based on this database, we identified overlapping differentially methylated CpG sites and neighboring genes across studies and performed functional enrichment and interaction network analyses. Based on these results, we highlighted pathways that may be involved in the development or progression of CVD through a mechanism that is associated with DNA methylation changes.

## Methods

### Systematic literature search

A systematic search was conducted in online databases “PubMed” (https://pubmed.ncbi.nlm.nih.gov) and “Cochrane Central Register of Controlled Trials” (https://cochranelibrary.com) (CENTRAL). Two search queries were used, which included references to DNA methylation and epigenetics in general, and a comprehensive listing of individual cardiovascular diseases based on the coding scheme of the International Classification of Diseases (ICD), version 10 (see Additional file 1: Text 1). All studies included in these databases between the respective database inception date and June 14, 2022 for PubMed and July 28, 2022 for CENTRAL were examined.

### Study selection process

Titles and abstracts of all identified articles were screened for eligibility by one scientist (MK) using the online tool Abstrackr (http://abstrackr.cebm.brown.edu) [7]. In case of any doubt about eligibility, the decision to include articles was discussed with a second scientist (VTC). Of all the articles remaining after screening, full texts were retrieved and assessed for suitability for systematic review. Eligibility criteria were discussed and established by an interdisciplinary team of epidemiologists, cardiologists, biologists and a biostatistician. As inclusion criterion, the article had to include data on DNA methylation with an association to cardiovascular disease (CVD) as an outcome or exposure. Articles that investigated DNA methylation in the context of CVD risk factors, without CVD as outcome or exposure, were excluded. Other exclusion criteria were: Irrelevant content, non-human samples, the publication was a review paper or the article was not available in full text in English. Reasons for inclusion or exclusion were recorded at each step (Additional file 2: Table 1).

**Table 1:** Study characteristics from *N* = 99 identified studies on cardiovascular epigenetics

	% of total	No. of articles
*Category*		
Heart	43.4	43
Vasculature	32.3	32
Cardiovascular disease (CVD)	12.1	12
Cardiovascular risk factors (CVRF)	8.1	8
Other	4.0	4
*Tissue*		
Blood	76.8	76
Cardiac tissue excluding ventricles	9.1	9
Cardiac ventricles	6.1	6
Aorta	5.1	5
Artery	3.0	3
Measuring method*		
Illumina bead-based 450 k array	46.5	46
Pyrosequencing	12.1	12
Illumina bead-based 850 k array	10.1	10
Whole-Genome Bisulfite Sequencing (WGBS)	4.0	4
Illumina bead-based 27 k array	3.0	3
Reduced representation bisulfite sequencing (RRBS)	2.0	2
Radiolabeling	2.0	2
Other	18.2	18
*Outcome*		
Ischemic Stroke	11.1	11
Atherosclerosis	10.1	10
Incident CVD	8.1	8
Incident MI	8.1	8
Incident HF	6.1	6
CAD	5.1	5
Incident Dilated cardiomyopathy	4.0	4
Acute coronary syndrome	3.0	3
AFIB	3.0	3
CHD	3.0	3
Other	38.4	38
Measuring type*		
Epigenome-wide methylation	59.6	59
Specific gene methylation	23.2	23
Global genome methylation	8.1	8
Epigenetic clock methylation	8.1	8
Specific CpG methylation	2.0	2
*Study location*		
Asia	38.4	38
Europe	36.4	36
North and South America	26.3	26
Australia	1.0	1
Sex**		
Female	54.0***	6986
Male	46.0***	5963
Age range**		
0–30 years	2.0	2
30–60 years	23.2	23
60–100 years	62.6	62
Sample size range**		
1–100	34.3	34
100–500	29.3	29
500–1000	15.2	15
1000–2000	7.1	7
2000–5000	10.1	10
≥ 5000	3.0	3

*Some studies used multiple measuring methods/measuring types

**Not all studies specified age range, sample size and sex of participants

***Percentages given considering n of reported male and female participants

### Quality control of the studies

The quality of each study was assessed using the following study quality assessment tools from the National Heart, Lung, and Blood Institute (NHLBI, Maryland, USA): quality assessment tool for observational cohort and cross-sectional studies, quality assessment of case–control studies, and quality assessment tool for case series studies (https://www.nhlbi.nih.gov/health-topics/study-quality-assessment-tools). The detailed quality assessment was recorded digitally.

### Data extraction

All study data were extracted and recorded digitally. Information collected included the following variables: study subjects, exposure, outcome, duration of follow-up, cohort, study design, DNA methylation measurement method, study location, sample size with details on cases and controls, tissue, sex and age. Detailed data were collected on individual CpG sites and genes reported in each study. This included beta estimates for the methylation level, p-values, standard errors, direction of methylation change and regression coefficients, where applicable. The collected information was organized in a database that was used for further analysis and is available in the Additional file 3: Table 2. CpG sites were mapped to genes using the publicly available 450 k [8] and 850 k [9] manifest files from Illumina (California, USA).

**Table 2:** Identified CpG sites and genes reported in relation to cardiovascular system

	No. of CpG sites		No. of mapped genes	
	Including/excluding methylation clocks		Including/excluding methylation clocks	
Overall reported	74,580	73,686	19,127	19,042
Reported in ≥ 2 studies	1452	1331	5805	5472
Reported in ≥ 4 studies	102	10	787	498

### Statistical and bioinformatics analysis

Aggregated values needed for the analysis were calculated and stored in the CpG database. Detailed calculations can be found in Additional file 4. CpG sites from each of these three “methylation clocks”: Horvath [10], Hannum [11] or GrimAge [12], were included separately in the CpG database. CpG sites from GrimAge [12] were not included in the publicly available version.

For analysis of curated gene sets, the Molecular Signatures Database (http://gsea-msigdb.org) was used. The gene sets were selected in an interdisciplinary discussion between biologists and cardiologists based on the greatest perceived relevance to CVD.

All analyses were performed in R version 4.0.0 [13]. For gene set enrichment analysis, the R package ClusterProfiler [14] and the online tool STRING [15] were used. A one-sided Fisher’s test was used to calculate the relevance of the overrepresentation of a particular gene set compared to the expected background. Unadjusted p-values below 0.05 report overrepresentation, with p-values considered as continuous measure of the evidence of a difference in this exploratory approach.

## Results

### Identified studies on DNA methylation in cardiovascular disease

**Fig. 1:**
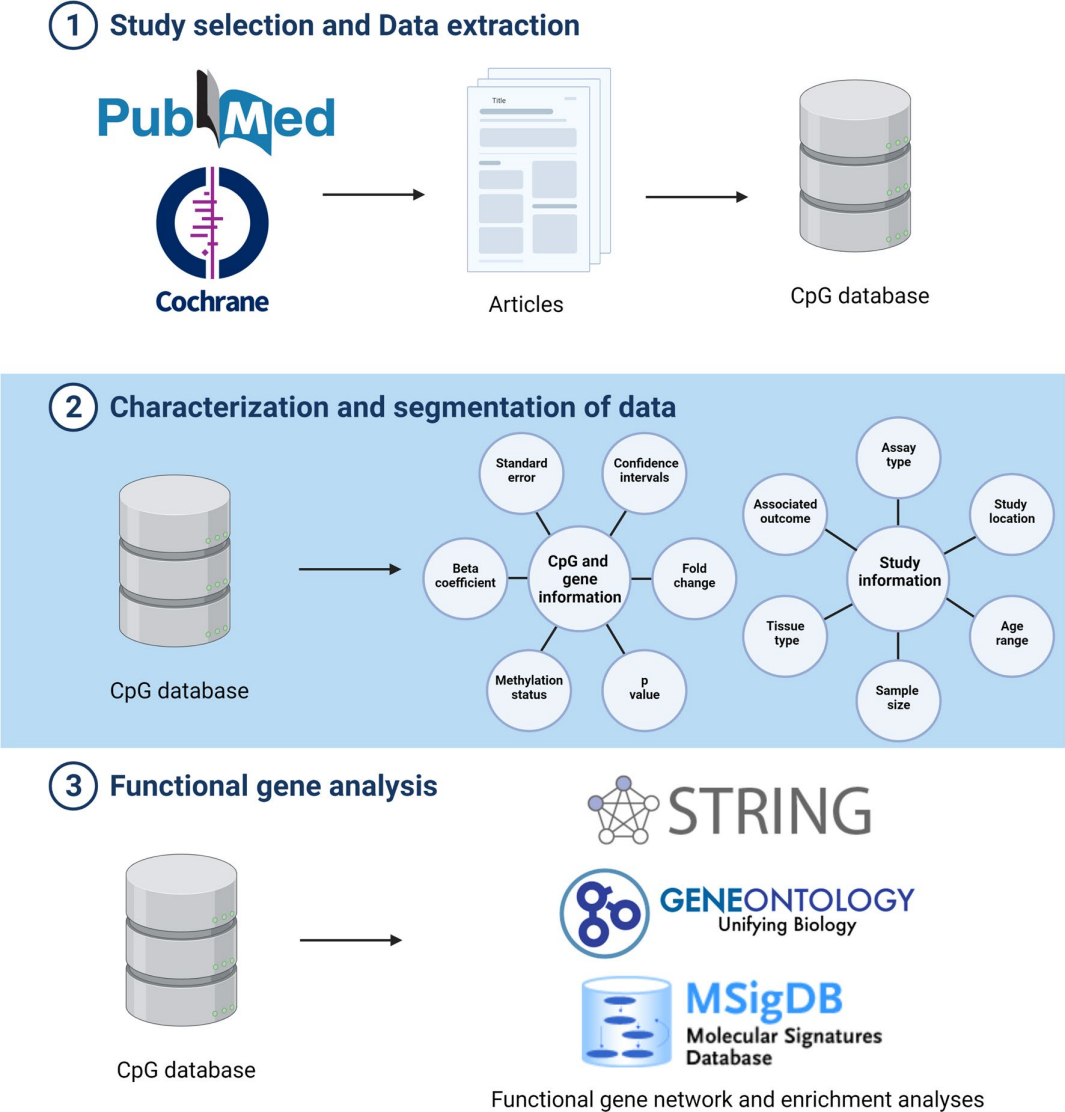
Workflow for the systematic review

An overview on the workflow of the systematic review is provided in Fig. 1.

**Fig. 2:**
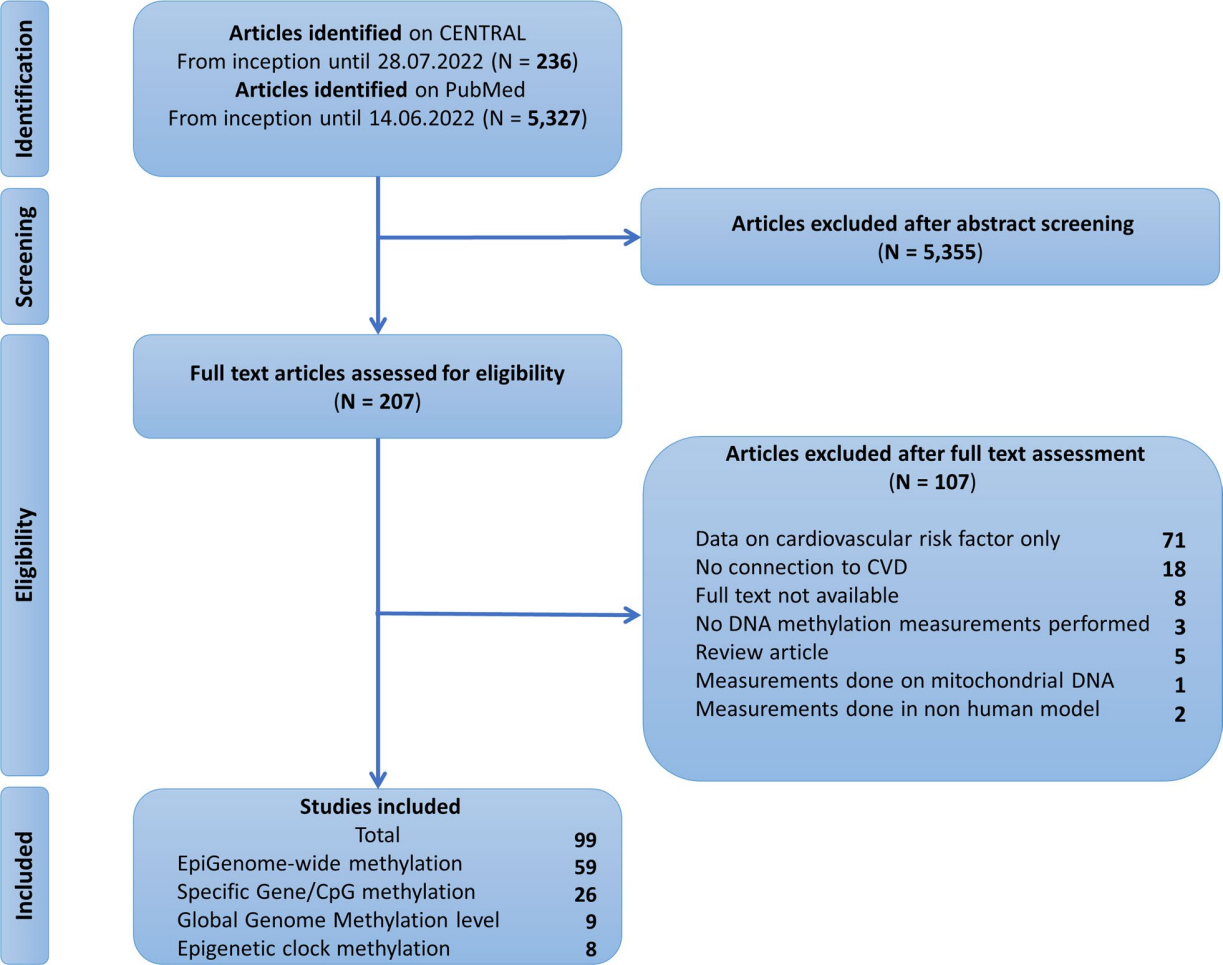
PRISMA flowchart. CVD—cardiovascular disease

Using the a priori defined search queries, a total of 5327 articles was identified in PubMed and 236 articles in CENTRAL. After irrelevant articles were removed, a total of 207 studies were assessed for relevance by reading the full text. A total of 99 articles [16–115] were considered eligible for systematic review (Fig. 2).

After grading for risk of bias, the majority of publications (*n* = 93) was assigned a study quality metric of “fair” using the NHLBI quality assessment tools, with two studies graded “poor” and four graded “good.” Eighty-five articles provided direct data comparing CpG sites and genes (beta-estimates of methylation level, p-value, standard errors, etc.), nine measured global DNA methylation (i.e., total hyper- or hypomethylation), and eight studies used a methylation-based clock without directly reporting summary statistics at the CpG site level.

A total of 87,827 individuals was included in the 99 selected studies. Across studies that reported the sex distribution of participants, the overall proportion of females was 54% and that of males was 46%. DNA methylation measurements were performed using a variety of assays including the Illumina Infinium® HumanMethylation 450 BeadChip (Illumina, California, USA), Infinium® HumanMethylation 850 BeadChip (Illumina, California, USA), Pyrosequencing, whole genome bisulfite sequencing, and others (Table 1). Study cohorts were located in four continents: Europe, Asia, North and South America, and Australia. Samples for DNA methylation measurements were extracted from a variety of tissues, with whole blood used in the majority of cases. Since the type of outcome varied widely across the studies, they were grouped into the following five main categories for analysis: ‘CVD’, ‘heart’, ‘vasculature’, ‘cardiovascular risk factor (CVRF)’ and ‘others’. Study designs were grouped as follows: ‘investigation of epigenome-wide methylation’, ‘global genome methylation’, ‘specific CpG site methylation’, ‘specific gene methylation’ and ‘epigenetic clock methylation’.

A meta-analysis of the collected data could not be performed since the endpoints, methods and reported estimates of the individual studies could not be sufficiently reconciled.

### Database of CpG sites associated with CVD

A CpG database was created by aggregating all CpG- and gene-related information from the collected studies together with three methylation clocks: Horvath [10], Hannum [11] and GrimAge [12]. The database includes 74,580 unique CpG entries (73,550 entries without the methylation clock “GrimAge”) and 19,127 gene entries (18,374 entries without the methylation clock “GrimAge”). For each CpG/gene, there is information on the outcome, follow-up, cohort, measurement method, location of study, sample size, tissue analyzed, age range and related publication. For each entry, additional quantitative information such as methylation beta value, p-value, z-score, standard error, false discovery rate, etc. is provided if the information was reported in the publication. The database can be found in Additional file 3: Table 2.

### Overlap between reported CVD-relevant CpG sites

Of the 85 studies that reported associations between CpG sites or genes and CVD, 78 reported CpG sites directly by identifier. A total of 1452 CpG sites were mentioned once or more in at least two of the publications, 441 CpG sites were mentioned once or more in at least three publications, and two CpG sites were mentioned in ≥ 6 publications (Table 2). The two latter CpG sites are cg01656216 near gene *ZNF438* (mentioned in [10, 22, 30, 60, 65, 108]) and cg03636183 near gene *F2RL3* (mentioned in [49, 69, 75, 79, 113], Table 3). These two CpG sites are also part of the methylation clocks. Three out of six papers mentioning cg01656216 [22, 30, 108] have vascular disease as an outcome, whereas the three other publications had investigated epigenetic age [10, 60, 65]. In the case of cg03636183, three out of six publications had coronary heart disease or myocardial infarction as the outcome [75, 79, 113], and the other three had investigated smoking or air pollution [12, 49, 69].

**Table 3:** Top 5 identified CpG sites and genes

	Reported in no. of studies	Annotated gene/CpG*	Associated traits**	Total no. of traits
*CpG*				
cg01656216	6	*ZNF438*	Heel bone mineral density, rheumatoid arthritis, multiple sclerosis, body height, pulse pressure measurement,	30
cg03636183	6	*F2RL3*	Ageing, platelet reactivity measurement	2
cg07553761	5	*TRIM59*	Aging, epigenetic status, type II diabetes mellitus, serum alanine aminotransferase measurement, eosinophil count, systolic blood pressure, eosinophil percentage of leukocytes	18
cg09809672	5	*EDARADD*	Lymphocyte count, autoimmune thyroid disease, PHF-tau measurement, hypothyroidism, response to simvastatin, type II diabetes mellitus, total cholesterol change measurement, response to fenofibrate	10
cg10281002	5	*TBX5*	PR interval, atrial fibrillation, electrocardiography, smoking status measurement, systolic blood pressure, smoking status measurement, diastolic blood pressure	35
*Gene*				
*PTPRN2*	11	cg01271455, cg05766510, cg22395765, cg23455837, cg19208749, cg16964025, cg14631503, cg08492145, cg22056595, cg25566285, cg04864441, cg02941085, cg20393882, cg25277638, cg14338779, cg13451497, cg09194449, cg09608412, cg17561365	Gut microbiome measurement, abnormality of refraction, adolescent idiopathic scoliosis, cognitive function measurement, mathematical ability, pathological myopia	52
*TEAD1*	10	cg25037165, cg19662708, cg19496491, cg18525873, cg06829681, cg04940570	Self-reported educational attainment, vital capacity, body height, neutrophil count, mathematical ability	34
*ZBTB16*	9	cg14042099, cg09890653, cg22768358, cg25101936, cg07631435, cg16246188, cg02042026, cg04628008, cg09593860, cg15309093. cg24452821, cg25009965.cg25577489	lymphocyte count. neutrophil-to-lymphocyte ratio. platelet-to-lymphocyte ratio, eczema, self-reported educational attainment	30
*F2RL3*	9	cg19006008, cg03636183, cg08067617, cg08200625, cg14021375	Ageing, platelet reactivity measurement	2
*HOXC4*	8	cg15233062.cg15700739, cg22198132, cg14108840, cg02491017, cg23697546, cg19186380, cg05408649, cg21493516, cg27138204, cg26201952, cg22370252, cg18473521, cg03146625, cg15648389, cg00243574, cg01683044, cg06714180, cg10005224, cg18843682, cg26035702	BMI-adjusted waist circumference. systolic blood pressure. smoking behavior, BMI-adjusted waist-hip ratio	44

Top 5 genes and Cpgs, ranked on the number of studies they were reported in are displayed

*CpG sites reported in investigated studies

**Association extracted from GWAS catalogue, top 5 traits by association count shown

### Overlap between reported genes

The collected 74,580 CpGs were mapped to 19,127 genes using the Illumina manifest files. Of these, 5807 genes were reported in at least 2 studies (Table 2). Two genes—*TEAD1* (TEA Domain Transcription Factor 1) and *PTPRN2* (Protein Tyrosine Phosphatase Receptor Type N2)—were reported most frequently (in ten and eleven articles, respectively). Both genes were mentioned in association with a variety of outcome events ranging from vascular to cardiac disease [11, 12, 22, 27, 30, 38, 44, 45, 54, 60–62, 65, 72, 92, 93] with *PTPRN2* being a predisposing factor for cardiac disease [12, 27, 30, 38, 44, 45, 54, 61, 72, 92, 93]. *TEAD1* is next to one CpG (out of 71 CpGs) underlying the Hannum clock [11] and *PTPRN2* was next to one CpG (out of 1030 CpGs) underlying the GrimAge clock [12].

### Enrichment analysis

**Fig. 3:**
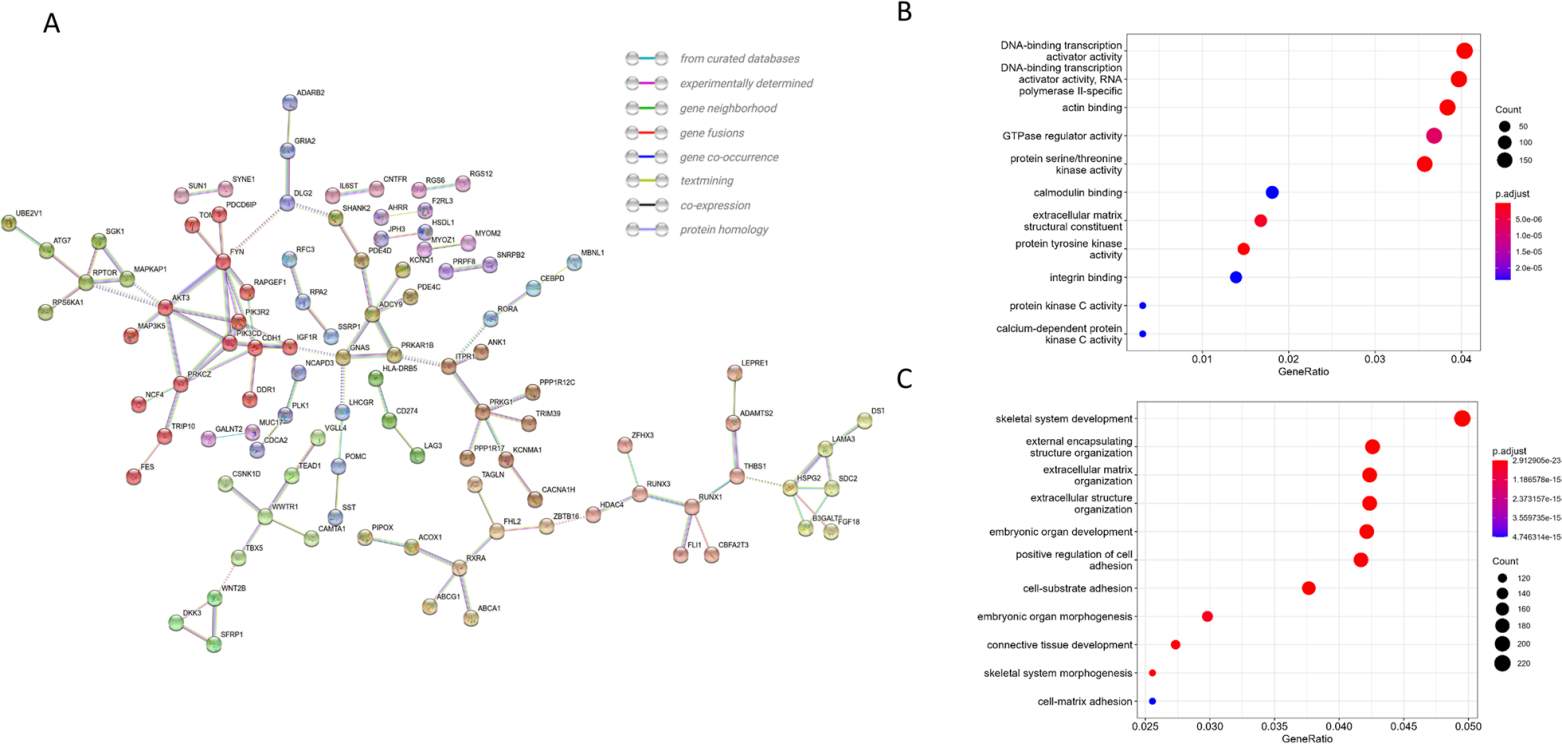
Gene enrichment analysis of identified genes. **A** Network analysis of genes occurring in ≥ 4 selected studies performed using STRING-db; genes without connections are hidden. **B** Enrichment analysis for the Gene Ontology databases “Molecular function” occurring in ≥ 2 selected studies. **C** Enrichment analysis for the Gene Ontology databases “Biological process” occurring in ≥ 2 selected studies

Gene enrichment analysis was performed for all 5,807 overlapping genes (Fig. 3) and then separately for the 5 outcome categories (‘CVD’, ‘Heart’, ‘Vasculature’, ‘CVRF’ and ‘Other’).

Of the gene IDs entered, 11.4% could not be mapped because their names were not available in the database. Analysis of the remaining 4,532 overlapping genes revealed enrichment of “DNA-binding transcription activator activity” (gene ratio, GR = 183/4,532, *q* = 1.65 × 10^–11^), “actin binding” (GR = 174/4,532, *q* = 6.97 × 10^–10^), “protein tyrosine kinase activity” (GR = 67/4,532, *q* = 7.98 × 10^–8^), “structural component of extracellular matrix” (GR = 76/4,532, *q* = 1.51 × 10^–6^), “GTPase regulator activity” (GR = 167/4,532, *q* = 6.40 × 10^–6^) and others for the molecular function of Gene Ontology (GO; Fig. 3-B). Enrichment analysis using the Gene Ontology database for biological processes for all overlapping genes showed enrichment for “skeletal system development” (GR = 221/4,462, *q* = 1.89 × 10^–23^), “extracellular matrix organization” (GR = 189/4,462, *q* = 1.89 × 10^–23^), “external encapsulating structure organization” (GR = 190/4,462, *q* = 1.89 × 10^–23^), and others (Fig. 3-C). The category “heart” showed similar enrichment to all categories combined with “DNA-binding transcription activator activity” being a top hit. The category “vascular” showed strong enrichment for the terms “actin binding” and “actin filament binding.” Genes assigned to the categories ‘CVD’, ‘CVRF’ and ‘other’ did not achieve a relevant enrichment. In STRING (Search Tool for the Retrieval of Interacting Genes/Proteins) analysis [15] with the confidence setting ‘high’, there was significant evidence for protein–protein interactions between the products of these genes (*p* = 0.003; Fig. 3-A). The average node degree was 0.735, and the average local clustering coefficient 0.223.

### Methylation of heart and vasculature

**Fig. 4:**
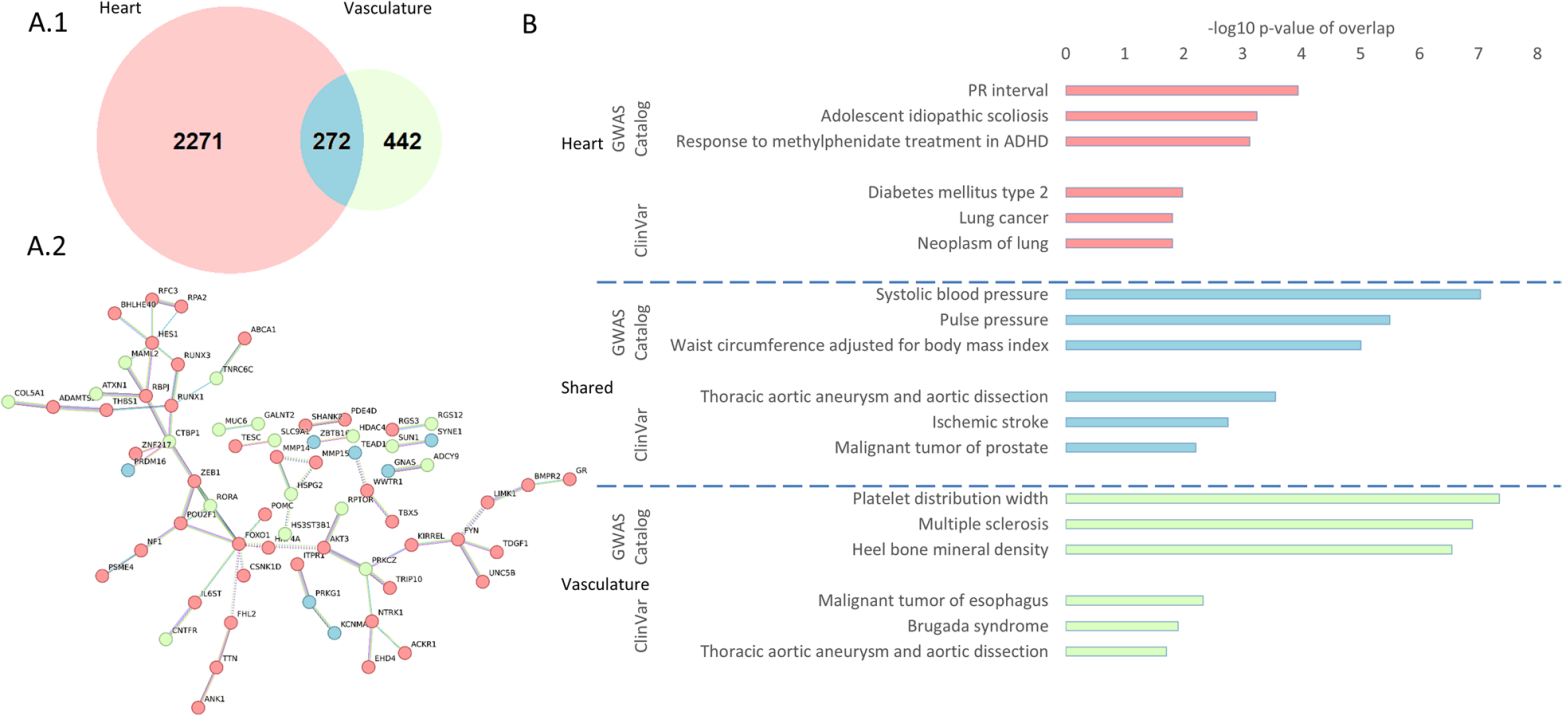
Methylation sites in heart and vasculature-related studies. **A1** Overlap of genes mentioned at least 2 times in Heart and Vasculature-related studies. **A2** Network analysis of genes occurring in Heart and Vasculature-related studies. Color coding corresponds to Figure **A** 1. **B** Gene enrichment analysis of the GWAS Catalog and ClinVar databases. Transformed p-values (One sided Fisher’s test) are shown. ADHD—Attention deficit hyperactivity disorder

When comparing the “heart” and “vasculature” categories, there is an overlap of 272 genes considering those reported in at least two studies. The compartment “heart” included 2,271 unique genes and the compartment “vascular system” included 442 unique genes (Fig. 4-A1 and A2). Analysis of gene enrichment using the GWAS Catalogue and ClinVar databases demonstrated that general cardiovascular disease-related terms are found in the “shared” category, while “heart” and “vasculature” specific genes have more disease-specific terms such as PR interval for “heart” or platelet distribution width for “vasculature” (Fig. 4-B).

### Overlap between identified genes and curated gene sets

**Fig. 5:**
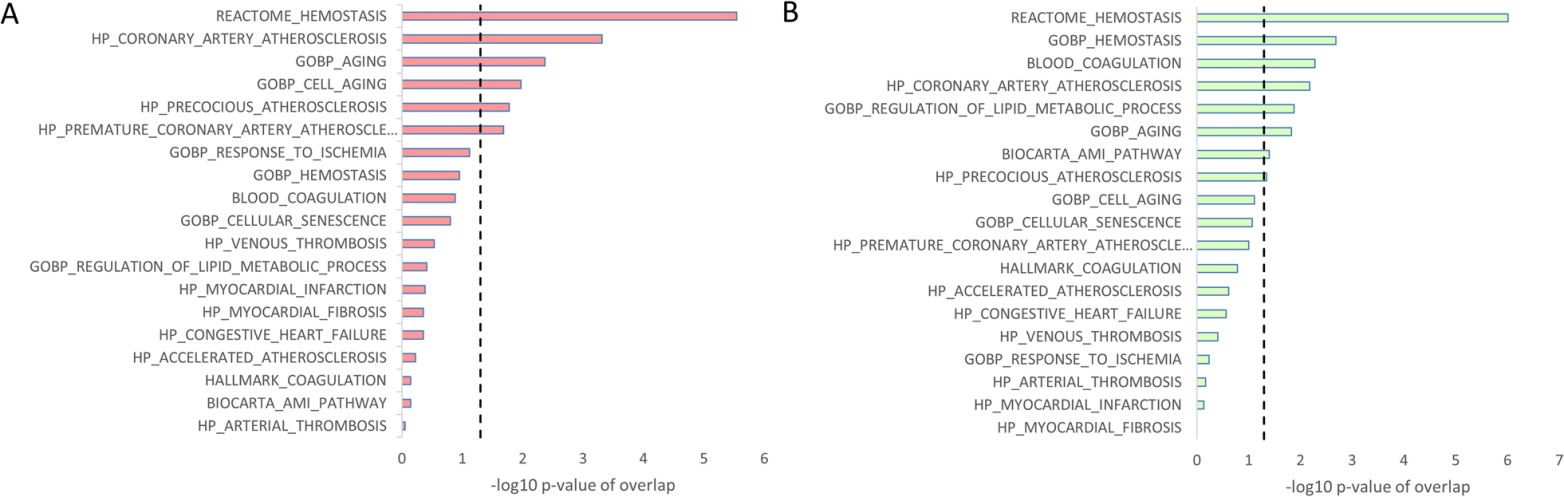
Overlap of identified genes and curated gene sets from the molecular signatures database (MSigDB). **A** Genes from Heart-related studies. **B** Genes from vasculature-related studies. Transformed *p*-values (One sided Fisher’s test) are shown in A. and B. AMI—acute myocardial infarction, HP—human phenotype, GOBP—gene ontology biological process

The overlap between pre-selected relevant curated gene sets from the Molecular Signatures Database (gsea-msigdb.org) and genes identified by several selected publications as related to CVD was analyzed: Significant gene set overlap was observed for the category “heart” (number of genes = 2486) with the genes from the datasets REACTOME_HEMOSTASIS (123/678, *p* = 2.9 × 10^–6^) and HP_CORONARY_ARTERY_ATHEROSCLEROSIS (14/44, *p* = 4.9 × 10^–4^, Fig. 5A).

For the “vasculature” category (n = 791), there was enrichment for the REACTOME_HEMOSTASIS (53/678, *p* = 9.8 × 10^–7^) and GOBP_HEMOSTASIS (7/47, *p* = 2 × 10^3^) gene sets (Fig. 5B). In the “CVD” category (*n* = 324), this was true for the HP_CORONARY_ARTERY_ATHEROSCLEROSIS gene set (3/44, *p* = 0.032). No relevant overlap was present with the categories “CVRF” and “Other.”

### Disease association

Previous studies have identified at least 48 genes from the category of “heart” that have been associated with coronary artery disease in general and CAD in patients with heart failure [22, 33]; Table 4). Other genes have also been found to be associated with specific cardiac conditions such as myocardial infarction [75, 79], cardiac hypertrophy [116], and cardiac remodeling [117]. In the “shared” category, 13 genes have been previously linked to weight loss [118], eight to congenital heart defects [119, 120], and individual genes have been associated with incident coronary heart disease [121] and myocardial infarction [122]. The “vasculature” category includes four genes that have been previously identified as related to chronic Chagasic cardiomyopathy [123], four to diabetes mellitus [124], and individual genes have been found to be associated with atherothrombotic stroke [125], cognitive function in CVD [126], and aortic dissection [127].

**Table 4:** Association of methylated genes with cardiovascular related diseases

	Genes	Associated disease	ICD –10 code	PMID
Heart	MIF,CAV2,ACSS2,JARID2,FAM212B,FRMD4A,CHM,TRPM6,PDSS2,NFIA,HTR1D,HS6ST3,DIS3,PARP4,SOX6,DAAM2,DOPEY2,MYO5A,PLA2G4E,HDAC9,MYO1H,ABCB1,GREM1,RPS6KA5,HYOU1	Coronary artery disease in patients with heart failure	I25*	32,618,141 [33]
	ST6GALNAC1,HOXA5,EMP1,SULF1,NGEF,SOST,HOXD4,TM4SF1,PLG,TMCO5A,WT1,IKZF1,ALDH1A3,ALX4,THSD4,PAX9,CEP170,S100A10,RNF207,GLRX,SH2D2A,ESR1,ELANE	Coronary artery disease	I25*	25,856,389 [22]
	F2RL3, ABTB2	Myocardial Infarction	I21.9	35,012,325 [75], 33,883,000 [79]
	ADAMTS2	Cardiac hypertrophy	I42.2	28,373,586 [116]
	ZBTB20	Cardiac remodeling	-	33,063,955 [117]
Shared	C7orf50, FBXL13, PRKCZ, KCNQ1, THBS1, PRDM16, DNMT3A, HOXA, HOXC4, TNXB, HOXB3, PTPRN2, SHANK2	Weight loss	R63. 4	25,651,499 [118]
	RUNX3, MYLK, GALNT2, TRAPPC9, PRDM16, NR2F2, HOXA3, HOXB3, AXIN2	Congenital heart defects (CHD)	Q24.9	31,186,048 [119], 30,760,879 [120]
	PTPRN2, TRAPPC9	Incident coronary heart disease	I25.10	34,627,379 [121]
	ACAP2	Myocardial infarction	I21.9	34,139,744 [122]
Vasculature	CD4, CCR5, CD8A, CXCR3	Chronic Chagasic Cardiomyopathy	B57. 2	31,087,713 [123]
	CD2, CCR5, CCR2, CD8A	Diabetes mellitus	E11.9	10,400,139 [124]
	ARHGEF10	Atherothrombotic stroke	I63.40	20,042,462 [125]
	CAMTA1	Cognitive function in adults with cardiovascular disease	-	21,951,953 [126]
	COL5A1	Aortic dissection	I71. 010	34,041,919 [127]

### Global DNA methylation level

The global methylation of DNA was investigated and reported by N = 9 studies. Six of them reported a moderate to large increase in global methylation level associated with the disease of interest: In a case–control study of coronary artery disease (*N* = 137 cases and *N* = 150 controls), global DNA methylation was quantified using radiolabeling with incorporation of [^3^H] dCTP (Deoxycytidine [3H] triphosphate tetra-sodium salt) [23]. Another study investigated global methylation levels in patients with and without acute coronary syndrome (n = 190) using an Enzyme-Linked Immunosorbent Assay (ELISA) [48]. Further studies examined *N* = 75 cardiomyopathy patients using immunoelectron microscopy [55], *N* = 286 subjects with self-reported history of physician-diagnosed myocardial infarction using the MethyLight method (Methylation-specific PCR [29, 128]), and in case–control studies using an ELISA-based kit, *N* = 20 patients who had undergone heart valve replacement surgery [95], and *N* = 44 individuals with coronary heart disease [83].

Only one study comparing 17 patients with atherosclerosis with 15 healthy individuals using radiolabeling reported global hypomethylation [56]. Two studies, one of 8 patients compared to 8 controls and another of 300 patients versus 300 controls, reported no change in global DNA methylation levels associated with atherosclerosis. These measurements were done with Illumina 450 k and Pyrosequencing, respectively [54, 73].

## Discussion

In this work, we systematically reviewed the current state of science in the field of cardiovascular epigenetics in humans using data from published clinical trials and summarized the methods and study results. CpG dinucleotides, genes and pathways were extracted from the compiled data and cross-referenced with publicly available databases that provide evidence that CpG methylation may be a potential factor in the development and progression of CVD. All the information collected was compiled into a novel publicly available database provided in the supplement that can serve as a basis for future research. Such an overview of data on methylated CpG sites and affected genes associated with CVD was not previously available.

The work included a large number of studies from four continents with a wide range of age groups, tissues and study designs. The studies predominantly used whole blood as tissue and applied the Illumina Infinium HumanMethylation 450 k as the method for measuring DNA methylation (DNAm). Whole blood is a relatively inexpensive and reliable source of DNAm information and in most cases it is difficult, for practical and ethical reasons, to obtain other tissues unless an invasive procedure is indicated. Although the use of whole blood is common in the case of CVD since diseases affecting the heart and vessels are significantly regulated via the blood and its components, multiple studies have consistently demonstrated that DNA methylation exhibits tissue-specificity [129–131]. While utilizing whole blood samples allows for improved comparability between studies, it also significantly limits the scope of investigation into the relationship between methylation in various tissues and specific diseases. Despite the tissue-specificity of DNA methylation, the methylomes of various tissues and cells reveal universal characteristics that are indicative of the overall health and age status of the organism, such as tissue-independent “methylation clocks” which can predict biological age and longevity [10, 12].

The widespread use of the Illumina 450 k array is not surprising, as the method is relatively cheaper compared to whole genome bisulfite sequencing and covers a large portion of the methylated genome. It is surprising, however, that only ten studies to date have used the newer 850 k method, even though it has been available for at least five years and is only slightly more expensive.

Using the data, this work investigated the CpG sites, genes, or pathways that have been described as differentially methylated in several studies on CVD. These could be important key sites for the link between DNA methylation and the disease. However, the field of cardiovascular epigenetics is still relatively young, and research activities to date have not yet converged on a standardized procedure. Although many scientists in the field have used similar study designs, the wide variety of measurement and analysis methods employed allows only limited comparison and prevents a more in-depth synthesis of existing knowledge. Evidence of this is the fact that CpG sites overlapping between studies were only identified in a maximum of six out of 99 studies. The fact that the same CpG sites were identified as differentially methylated in studies of different diseases not only confirms that there is a strong link between DNA methylation as a global process and CVD, but also supports the hypothesis that methylation of specific CpG sites is also likely to be disease-relevant. This is further supported by the examination of CpG-annotated genes. CpG-annotated genes that are investigated in studies pertaining to the heart and vasculature typically exhibit associations with specific diseases related to those tissues, while genes that are shared between them are linked to more general cardiovascular disease terms and conditions. However, there is an urgent need to investigate the effects of individual CpG sites on the phenotype in more detail, as most authors only describe the effects at the level of the gene or gene region where the CpG site is located. It is also interesting to note that some of the differentially methylated CpG sites identified in this analysis are also part of methylation clocks. This is evidence that CVD is the most important life-limiting factor in the population, but may also indicate a more specific link between epigenetic ageing processes and CVD. Indeed, several studies have reported significant associations between methylation clocks and CVD [12, 132, 133].

Looking at the most frequently observed methylated genes in the studies, whether directly mentioned or derived from the CpG sites analyzed, many of them exhibit properties specifically related to cardiovascular processes such as atherosclerosis, hemostasis, and coagulation. The association of CVD with the gene level has already been documented in large GWAS studies [134]. The association of the CpG dinucleotides with CVD was also confirmed when comparing the identified genes with curated gene sets. The results of enrichment analyses with pathomechanistically relevant processes such as coagulation also underscore this relationship.

As mentioned above, the many different methods for measuring the methylation of DNA make analyses that aim to summarize or build on existing knowledge difficult. This methodological heterogeneity is due to multiple factors. On the one hand, technologies are constantly evolving and new methods are arising every year; on the other hand, it takes time to introduce and implement new methods in clinical trials. In addition, differences between studies, e.g., in terms of geographical location, ethnic composition of cohorts, sex distribution, endpoints analyzed and statistical methods used, make it difficult to synthesize the evidence. In perspective, there is a great need for further studies and research to investigate the clinical impact of CpG methylation on molecular, subclinical, and clinical parameters to better understand the association between DNAm and CVD.

## Conclusion

This review highlights the significant relationship between DNA methylation and CVD in humans. Numerous CpG methylation sites, genes and pathways have already been discovered that may play an important role in this context. Methylated CpG sites identified in heart and vasculature-related disease belong to genes with distinct functions known to be important in CVD. Orthogonal evidence from genome-wide association studies confirms that these genes have downstream impact on the cardiovascular phenotype, ranging from vascular markers such as blood pressure to cardiac function. The open-access database provides an overview of the identified CpG sites and the associated results from 99 studies. This will facilitate access to this information for future research in the field and support research in cardiovascular epigenetics.

## Supplementary Information

**Additional file 1. Text 1**. Detailed calculations used in figures and tables.

**Additional file 2. Table 1**. Study characteristics of the published studies analyzed.

**Additional file 3. Table 2**. Database of CpG sites reported to be associated with development and progression of cardiovascular disease.

**Additional file 4**. Detailed search query for the systematic review.

## Data Availability

All data collected, generated, or analyzed as part of this study, except for data from the GrimAge methylation clock, are included in this published article, or available as additional files.

## Abbreviations

AMI: Acute myocardial infarction
ACS: Acute coronary syndrome
CpG: Cytosine-phosphate-guanine
CVD: Cardiovascular disease
CVRF: CVD risk factor
DNA: Deoxyribonucleic acid
DNAm: Deoxyribonucleic acid methylation
GO: Gene ontology
GOBP: Gene ontology biological process
HP: Human phenotype
PRISMA: Preferred reporting items for systematic reviews and meta-analyses
