# Supplementary material for: DNA methylation and cardiovascular disease in humans: a systematic review and database of known CpG methylation sites
Source: Clin Epigenetics. 2023 Mar 30;15:56. doi: 10.1186/s13148-023-01468-y (PMC10061871; doi:10.1186/s13148-023-01468-y)
Supplement: Supplementary file 1 — Additional file 1. Text 1. Detailed calculations used in figures and tables. [file 13148_2023_1468_MOESM1_ESM.docx]

PubMed query:

("methylation"[title/abstract] OR "DNAm"[title/abstract] OR "epigenetic"[title/abstract] OR "epigenetics"[title/abstract] OR ("epigenetic clock") OR "DNA methylation"[title/abstract]) AND ("cardiovascular"[title/abstract] OR "CVD"[title/abstract] OR "heart failure"[title/abstract] OR "atrial fibrillation"[title/abstract] OR "coronary artery disease"[title/abstract] OR "peripheral artery disease"[title/abstract] OR "myocardial infarction"[title/abstract] OR "stroke"[title/abstract] OR "cerebrovascular"[title/abstract] OR "venous thromboembolism"[title/abstract] OR "pulmonary embolism"[title/abstract] OR "deep vein thrombosis"[title/abstract] OR "aneurysm"[title/abstract] OR "sudden cardiac death"[title/abstract] OR "arrhythmia"[title/abstract] OR "transient ischemic attack"[title/abstract] OR "cardiomyopathy"[title/abstract] OR "atherosclerosis"[title/abstract] OR "varicosis"[title/abstract] OR "chronic venous insufficiency"[title/abstract] OR "post-thrombotic syndrome"[title/abstract] OR "thrombophlebitis"[title/abstract] OR "valvular"[title/abstract] OR "pulmonary hypertension"[title/abstract] OR "atrial flutter"[title/abstract] OR "thrombosis"[title/abstract]) AND "english"[language]

CENTRAL query:

("methylation":ti,ab,kw OR "DNAm":ti,ab,kw OR "epigenetic":ti,ab,kw OR "epigenetics":ti,ab,kw OR ("epigenetic clock") OR "DNA methylation":ti,ab,kw) AND ("cardiovascular":ti,ab,kw OR "CVD":ti,ab,kw OR "heart failure":ti,ab,kw OR "atrial fibrillation":ti,ab,kw OR "coronary artery disease":ti,ab,kw OR "peripheral artery disease":ti,ab,kw OR "myocardial infarction":ti,ab,kw OR "stroke":ti,ab,kw OR "cerebrovascular":ti,ab,kw OR "venous thromboembolism":ti,ab,kw OR "pulmonary embolism":ti,ab,kw OR "deep vein thrombosis":ti,ab,kw OR "aneurysm":ti,ab,kw OR "sudden cardiac death":ti,ab,kw OR "arrhythmia":ti,ab,kw OR "transient ischemic attack":ti,ab,kw OR "cardiomyopathy":ti,ab,kw OR "atherosclerosis":ti,ab,kw OR "varicosis":ti,ab,kw OR "chronic venous insufficiency":ti,ab,kw OR "post-thrombotic syndrome":ti,ab,kw OR "thrombophlebitis":ti,ab,kw OR "valvular":ti,ab,kw OR "pulmonary hypertension":ti,ab,kw OR "atrial flutter":ti,ab,kw OR "thrombosis":ti,ab,kw)
